# Human Mesenchymal Stromal Cell-Mediated Immunoregulation: Mechanisms of Action and Clinical Applications

**DOI:** 10.1155/2013/203643

**Published:** 2013-09-29

**Authors:** Akaitz Dorronsoro, Jon Fernández-Rueda, Karoline Fechter, Izaskun Ferrin, Juan Manuel Salcedo, Emma Jakobsson, César Trigueros

**Affiliations:** Mesenchymal and Hematopoietic Stem Cell Department, Fundación Inbiomed, Foundation for Stem Cell Research, Paseo Mikeletegi 81, 20009 San Sebastián, Spain

## Abstract

Mesenchymal stromal cells (MSCs) are multipotent cells found in connective tissues that can differentiate into bone, cartilage, and adipose tissue. Interestingly, they can regulate immune responses in a paracrine way and allogeneic MSCs do not elicit immune response. These properties have encouraged a number of clinical trials in a broad range of regenerative therapies. Although these trials were first focused on their differentiation properties, in the last years, the immunosuppressive features have gained most of the attention. In this review, we will summarize the up-to-date knowledge about the immunosuppressive mechanisms of MSCs in vivo and in vitro and the most promising approaches in clinical investigation.

## 1. Introduction

Mesenchymal stromal cells (MSCs) are found in a variety of tissues, although bone marrow represents the most common source for research and clinical purposes. These cells are multipotent progenitors that have the capacity to differentiate into multiple lineages such as bone, cartilage, and adipocytes [1–3]. MSCs have received renewed interest in the last five years, particularly due to their ability to modulate the immune response. This property, in combination with the facts that they are not immunogenic and preferentially home to damaged tissue, makes them good candidates for a therapeutic approach of cell-based therapy for a wide range of autoimmune disorders [4–6]. Currently, there are a large number of ongoing clinical trials employing MSCs as immunomodulators.

MSCs have been shown to regulate the activity in a range of effector cells involved in both innate and adaptive immunities. The crosstalk between MSCs and the cells of the immune system leads to an increased production of several soluble immunomodulatory factors. Despite identification of many of these factors, the mechanism behind MSCs immunomodulation is not fully understood. However, the inflammatory environment and in particular the immune cells involved in each phase of the immune response are likely to be the critical determinants of the regulatory process. The immunosuppressive ability of MSCs is not constitutive but rather is induced by crosstalk with cells of the immune system [7–10]. Therefore, different inflammation status might lead to distinct immunomodulatory responses. This is a fundamental concept that could determine future clinical settings: treatment dose, timing, and frequency of administration, as well as the choice of the source of MSCs.

## 2. MSC-Mediated In Vitro Immunosuppression

The ability of MSCs to modulate several processes of the immune system in vitro has been intensively studied in the last years. MSCs have a broad range of target immune effector cells and are able to inhibit key functions of innate and adaptive immune cells during inflammatory responses. The exact mechanisms by which MSCs are able to regulate immune functions are still not fully understood. However, while the requirement of cell-to-cell contact is not clear, a number of soluble factors involved in the process have been identified.

The most studied soluble molecules and cytokines secreted by MSCs and involved in immunosuppression are indoleamine 2,3-dioxygenase (IDO), prostaglandin E2 (PGE2), transforming growth factor-beta (TGF-*β*), hepatocyte growth factor (HGF), and interleukin-10 (IL-10). Furthermore, in the past few years, the implication of other molecules like human leukocyte antigen-G (HLA-G5) has been shown to be associated with the immunomodulatory properties of MSC. 

### 2.1. T Cells

MSC-mediated inhibition of T cell proliferation has been largely described. In vitro, MSCs can inhibit T cell proliferation regardless of the signaling pathway stimulated in the lymphocytes (i.e., alloantigen-, mitogen-, or anti-CD3/anti-CD28-mediated stimulation) [11, 12]. This immunomodulation does not require antigen match, as it has been shown to have similar efficiencies in cocultures of MSCs and T cells from the same donor or from different donors. Upon coculture, MSCs induce a G0/G1 checkpoint arrest in T cells, but whether this leads to a permanent arrest or if they can resume proliferation once separated from MSCs remains unclear [12, 13].

The mechanisms by which MSCs are able to mediate immunosuppression of T cells are diverse and complex, and several secreted effector molecules have been linked to this process. Among them, IDO, PGE2, TGF-*β*, and HGF have been described to play a major role.

IDO is the first and rate-limiting enzyme in tryptophan metabolism catalyzing the breakdown of tryptophan to N-formylkynurenine. It is known that both tryptophan starvation and the presence of N-formylkynurenine induce proliferative arrest of T cells [14, 15]. In MSCs, IDO is not constitutively expressed, but upon stimulation with inflammatory cytokines, mainly by IFN-*γ*, the transcription is increased significantly [16]. Once IDO is expressed in the MSC, this enzyme catalyzes the breakdown of tryptophan and induces the immunomodulative effect of MSCs on T cells [17].

PGE2 is synthesized from fatty acids by cyclooxygenases 1 and 2 (COX-1, COX-2), and its immunosuppressive effects on T cells have been largely studied. PGE2 reduces interleukin-2 (IL-2) and interferon-gamma (IFN-*γ*) secretion and increases cyclic adenosine monophosphate (cAMP) levels, leading to the inhibition of T cell proliferation [18]. PGE2 is constitutively produced by MSCs and proinflammatory factors increase its secretion. Chemical inhibition of COX-2 in MSCs and T cell cocultures reduces PGE2 levels, resulting in a partial impairment of MSC-driven immunosuppression [19]. 

The role of TGF-*β* in immunosuppression is still not fully known. TGF-*β* is constitutively secreted by MSCs, and its expression is notably increased in the presence of peripheral blood mononuclear cells (PBMCs). Although different TGF-*β* isoforms have been linked to immunoregulation, as shown by the addition of neutralizing antibodies to cocultures or mixed lymphocyte reaction (MLR) alone [12, 20], other publications rule out this function [21]. 

To which extent HGF, constrictively expressed in MSC [16], plays a role in MSC-mediated immunosuppression still remains unclear. While some authors point out a role for this factor alone or demonstrating additive effect together with TGF-*β* [12, 20], others discard its function in immunoregulation, rather attributing its existence in cocultures to its production by lymphocytes [21].

Finally, HLA-G5, a nonclassical HLA class I molecule, has first been shown in the maternal tolerance to the fetus by mediating inhibition of NK cell cytotoxicity. Since then, its role in immunoregulation has been described in various types of pathological conditions such as viral infections, tumors, autoimmune diseases, and solid organ transplantation [38]. HLA-G5 is expressed on and secreted by adult MSCs, and its expression is linked to the immunosuppressive effects of MSCs on activated T cells via a mechanism including CD4^+^CD25^+^Foxp3^+^ regulatory T cells and IL-10 [39–41]. 

### 2.2. Natural Killer Cells

The relationship between MSCs and NK cells is ambiguous, and the mechanisms by which MSCs regulate inflammatory functions of NK cells are not well understood. On the one hand, freshly isolated NK cells fail to attack MSCs, while in contrast in vitro preactivated NK cells acquire this ability [42]. On the other hand, MSCs can inhibit proliferation, cytokine secretion and cytotoxicity of NK cells. PGE2 and TGF-*β* secretion, by MSCs has been linked to antiproliferative effects and reduction in cytokine production. Data suggest that cell-to-cell contact is also involved in this type of immunomodulatory effect. Several groups have demonstrated the ability of MSCs to impair the proliferation of NK cells activated by IL-2 and interleukin-15 (IL-15). Secretion of IFN-*γ*, tumor necrosis factor-alpha (TNF-*α*), and IL-10 by activated NK cells is also reduced by MSCs in vitro, in cell-to-cell contact conditions and independently of the contact mechanisms. Moreover, MSCs are able to inhibit the cytotoxic activity of NK cells when they are targeted against major histocompatibility complex-I positive (MHC-I), but not MHC-I negative tumors [19, 43, 44].

### 2.3. B Cells

The effect of human MSCs on B cells mainly depends on several factors linked to B cell biology, the state of differentiation, and the kind and strength of stimulus given to the cells. Furthermore, results depend on dissection of viability, proliferation, and differentiation of B cells and show strong dependence on methodological differences. While some studies show that MSCs inhibit B cell proliferation, others claim that this effect is not statistically significant, or even that MSCs stimulate B cell proliferation [45–48]. The same is true for immunoglobulin (Ig) secretion studies where results range from inhibition to increase [45] of secretion of IgA, IgG, or IgM by MSCs [46].

### 2.4. Monocytes

The role of MSCs in immunoregulation of monocytes is considered to affect both different maturation steps and their function as antigen-presenting cells (APCs). First, MSCs impair the differentiation and maturation towards effector dendritic cells (DCs). They can induce a reduction in the expression of CD1a, CD40, CD80, CD86, and MHC-II in monocytes along macrophage differentiation and impair the induction of CD40, CD83, and CD86 during the following maturation [49, 50]. Second, in mature DCs, some data suggest that MSCs interfere with their phagocytic function and ability to activate T cells [51]. Moreover, cytokines like interleukin-6 (IL-6), PGE2, and macrophage-colony stimulating factor (M-CSF) [49, 51] secreted by MSCs can in turn modulate the cytokine profile of DCs. This leads to stimulation of DCs to secrete the anti-inflammatory cytokine IL-10 and to reduce the secretion of interleukin-12 (IL-12) and TNF-*α*, creating an anti-inflammatory environment and furthermore influencing the function of other immune cells [19].

### 2.5. Neutrophils

The two main effects of MSCs on neutrophils are an increase of their lifespan and inflammatory activity. Consistent results show a reduction of the spontaneous apoptosis rate in both resting and activated neutrophils mediated by MSCs secreting IL-6. Apoptosis in neutrophils is positively linked to production of reactive oxygen species (ROS). Hence, as MSCs have been shown to inhibit ROS production, they lead to decrease inflammatory activity and reduced onset of respiratory burst of neutrophils [52–54].

## 3. Clinical Trials

To date, more than 290 clinical trials involving infusion or transplantation of MSCs have been registered at clinicaltrials.gov, the largest part of which depend on the immunomodulatory properties of MSCs. Most of these trials have not been completed yet, but data collected up to now support the biosafety of MSC transplantation in humans. So far, none of them has reported significant pathological incidences related to transplanted cells. Even if generally they have shown clinical benefits below the expected and immunoregulatory mechanisms are not fully understood, there is a general agreement that therapies based on immunomodulatory features of MSCs aiming to treat a number of immunological disorders that currently have no effective treatments are promising.

Experiments aiming to restore damaged tissues relied first on the ability of MSCs to give rise to mesodermal lineages and eventually also on their immunomodulatory and trophic effects. Despite the success of preclinical studies with MSCs in tissue repair [55] and the fact that most phase I clinical trials have not shown any biosafety issues, further trial phases have obtained modest outcomes. Patients with ischemic stroke have been subjected to a phase I clinical trial, in which MSCs were infused to patients aiming to regenerate infarcted tissue. MSC-treated group showed a slightly better recovery than control group [56] in terms of Modified Rankin Scale values after a 3-year follow-up. Several clinical trials have been initiated since then, including a phase II/III trial in Yonsei University in Seoul (clinicaltrials.gov NCT01392105). Also, MSC-based Prochymal developed by Osiris Therapeutics is currently been tested in a myocardial infarction phase II study (clinicaltrials.gov NCT00877903).

Crohn's disease and ulcerative colitis are inflammatory bowel diseases in which progression impaired immune function plays a key role. Phase I studies have been performed by systemic injection of MSCs in patients that did not respond to conventional treatments; their outcome varies from discrete improvements in Crohn's disease activity index (CDAI) [57] to adverse effects due to a mild allergic reaction to the DMSO used for the cryopreservation of the MSCs [58]. Application of Prochymal in Crohn's disease treatment has also shown to be safe, and a phase III clinical trial has been started (clinicaltrials.gov NCT01233960). 

Crohn's disease often results in the formation of perianal fistulae, which can also result from other inflammatory diseases, such as cryptoglandular disease. A phase I trial involved the local transplantation of autologous MSCs from lipoaspirates in five patients to test the feasibility and safety of the system. 75% of treated fistulae were effectively healed, with no adverse effects reported [59]. A later phase II trial in perianal fistulae both with Crohn's disease origin or cryptoglandular origin was conducted in 49 patients using MSCs from lipoaspirates, with an effectiveness of 71% and a recurrence of 17.6% after one-year follow-up regardless of the origin of the fistula [60]. However, in a later multicenter, phase III study focused on complex fistulae not associated with Crohn's disease, the same authors did not find significant differences between the control and MSC-treated groups [61].

Multiple sclerosis (MS) is a neurodegenerative inflammatory disease in which antibodies are produced against myelin sheaths of the brain and spinal cord neurons, leading to a wide variety of neurological disorders. Despite the efficiency of MSC treatment in rodent models of experimental encephalomyelitis (Table 1), an induced disease that mimics MS, clinical trials have not obtained positive results so far. As MS is a complex disease with prolonged onset, some trials have focused their endpoints on specific symptoms as a model of a wider effect. A phase I trial with intrathecally delivered autologous MSCs reported improvement of some visual features [62]. In a more recent phase II clinical trial, MS patients in whom disease involved degeneration of the optic nerve were infused intravenously with autologous MSCs. This trial reports modest improvements in the area of the optic nerve and some visual features, such as visual acuity and minimum angle of resolution [63]. 

It is widely believed that immune response plays also a crucial role in the development of amyotrophic lateral sclerosis (ALS). ALS is an incurable neurodegenerative disease that progresses rapidly impairing motor neuron function. MSC-based cell therapy has emerged as a promising approach to treat these neurological diseases, due to, on the one hand, their ability to support tissue regeneration and local stem cell stimulation through trophic effects and, on the other hand, their immunomodulatory properties. A third feature of MSCs has been proposed. It is very controversial, which claims that MSCs might have transdifferentiating capacity towards neural lineages [64–66]. 

A phase I clinical trial was performed in ten ALS patients by injecting MSCs in cerebrospinal fluid. Patients showed no symptoms of adverse effects, although the effectiveness of the treatment could not be assessed [67]. A second phase I study, involving both ALS (*n* = 19) and MS (*n* = 15) patients, was performed with intrathecally or intravenously delivered magnetically labeled MSCs. Mild adverse effects, including transient fever and headache, were attributed to the injection. Data suggested that a MSCs population had migrated to meninges, subarachnoid space and spinal cord. Regarding immunomodulatory outcome, MSC transplantation resulted in an increase in CD4^+^CD25^+^Foxp3^+^ regulatory T cells and a downregulation of activated lymphocytes and antigen-presenting cells [68]. These treatments have shown no biosafety issues, so several phase II trials have been initiated.

Diabetes is also a promising target for MSC-based therapies, due to its local, autoimmune nature and its high prevalence and severity. MSCs' ability to suppress autoimmunity against islets, help damaged islet regeneration, and enhance survival of engrafted islets in mouse diabetes models (Table 1) has encouraged the initiation of a number of phase I-II clinical trials, most of which are still ongoing. In the clinicaltrials.gov database, more than 20 trials involving MSCs, adipose-derived cells, or Prochymal for treatment of diabetes are listed.

Graft-versus-host disease (GVHD) is, at least in theory, one of the most suitable candidates for MSC-based applications. In its acute form, this systemic immune reaction prompted by grafted immune cells can be very severe and is often refractory to classical immunosuppressive treatments. The treatment of GVHD by MSC transplantation has successfully overcome phase I and II clinical trials. A pioneer compassionate study involving only one patient showed a complete response after 1 year [69]. In a later compassionate use study, Prochymal was delivered to twelve pediatric patients suffering severe acute GVHD refractory to immunosuppressive treatments. Severe acute refractory GVHD has been reported to have an overall 2-year survival of 5%–30% [70]. Overall, infusions were well tolerated and no acute toxicity was observed. Regarding effectiveness, even if some patients had only partial response to treatment, the overall surviving rate after a median of 611 days rose up to 42% (five patients) [71]. Another phase II study has been performed with this product in grade II–IV patients, with promising response and survival rates and no adverse effects reported. In this study, conventional steroid and mycophenolate mofetil therapy was supplemented with Prochymal treatment; 77% of patients achieved a complete response (CR), while steroid therapy alone usually leads to a CR rate of 10–35%. In patients achieving CR the survival rate at day 90 was 88% [72]. Currently, GVHD treatment using Prochymal is being tested in two phase III studies by Osiris, one of them involving newly diagnosed patients (NCT00562497) and the other one aiming to treat steroid-refractory GVHD (NCT00366145). Preliminary results announced by press release include a slight improvement in response and durable CR rates specifically in liver GVHD and gastrointestinal GVHD steroid-refractory patients and an overall trend of better response rates in pediatric patients [73]. In 2012, Health Canada and Medsafe New Zealand approved Prochymal as their first stem cell therapy for GVDH in children under 18 [74]. Besides, a phase II study using MSCs derived by the European Group for Blood and Marrow Transplantation ex vivo procedure involved 55 severe acute GVHD patients and showed an improvement of 2-year survival rate (53% versus 26% of uninfused patients), in the absence of side effects [75].

Although GVHD clinical trials have pioneered the use of MSC-mediated immunosuppression to avoid rejection of allografts, increasing numbers of MSC-based clinical trials are being initiated aiming to improve transplanted solid organ tolerance. Experiments in mice have shown that MSCs can increase immune tolerance for allografts (Table 1), and several clinical trials have been started since then. Mostly, MSCs are regarded as a strategy to reduce immunosuppressant pharmacotherapy doses, as transplantation success is often linked to drug-induced secondary effects. A pilot study has recently suggested that MSC delivery after kidney transplant is safe and allows reduction of tacrolimus dose to a half [76]. Another study has suggested that MSC treatment also allows reduction of the dose of calcineurin inhibitors (CNI), which are administered to reduce acute rejection reactions. In that clinical trial, patients receiving lower CNI doses plus MSC infusion had similar rejection rates after kidney transplant, and slightly better renal function recovery [77]. Furthermore, several phase I-II clinical trials with MSCs are ongoing in liver transplant. However, the outcomes are not available yet.

Despite the very optimistic results obtained in mouse models, MSC-based preliminary clinical trials have not fully met the expectations. The lack of obvious outcomes in clinical trials may be the result of specific human MSCs features and MSC-niche interactions and must be further addressed by analyzing these factors in human contexts. Indeed, there is renewed interest in studying these specific interaction processes in order to boost the therapeutic effect of MSCs. Examples of this effort are genetic manipulation of MSCs or in vitro priming of MSCs cultures, which is discussed below.

## 4. Priming MSC-Mediated Immunosuppression 

The ability of MSCs to modulate the activity of surrounding cells is not constitutive but rather requires activation by signals from a proinflammatory environment [20]. This process, termed priming or licensing, is very complex, and little is known about all the factors and signaling pathways involved [9]. In the last years, several papers have been published revealing the role of interleukin-1 (IL-1), IFN-*γ*, and TNF-*α* in this process [16, 20, 78].

Although the precise signaling pathway involved in the priming by IFN-*γ* is unknown, different authors describe that IFN-*γ* activates the transcription and synthesis of IDO-1 and increases expression levels of HGF and TGF-*β* in MSCs [16, 78]. In addition, IFN-*γ* stimulation of MSCs renders them resistant to activated NK-mediated attack, thereby allowing them to survive in the inflammatory environment [44]. In mouse models of colitis, the stimulation of human MSCs in vitro with IFN-*γ* before the transplantation yields better curative results [79]. These experiments reveal that priming by IFN-*γ* can increase the therapeutic effect of transplanted MSCs and points out a concept that should be taken into consideration in the design of clinical trials. However, it has been described that low concentrations of IFN-*γ* promote antigen-presenting cell function on MSCs and exert a proinflammatory effect [80]. This data should be evaluated carefully if this methodology is going to be implemented in future clinical trials. 

IL-1*β* is another cytokine linked to MSC priming [20]. Secreted by macrophages in the inflammatory environment, this cytokine not only activates the secretion of immunosuppressive factors in MSCs but also increases migration of MSCs and the secretion of leukocyte-chemotactic factors. Unlike the stimulation of MSC with IFN-*γ*, it is known that IL-1*β* exerts its effect partially through the activation of NF-*κ*B signaling [81]. In this sense, it has been published that TNF-*α*, a proinflammatory cytokine secreted by T cells, NK cells, and macrophages, induces the expression of various proteins related to immunosuppression and increases the migration of stromal cells [82–84]. 

The fact that these proinflammatory cytokines and signaling pathways can also activate MSC-mediated immunosuppression may seem paradoxical. When the inflammatory reaction overshoots, the same signals that are normally involved in inflammation prime the MSCs and activate their immunomodulatory properties as a negative feedback safety loop. In this regard, the overload of proinflammatory cytokines is precisely the necessary step that activates immunosuppression in MSCs in order to avoid a nondesired immunosuppression due to resident stromal cells. Moreover, in the absence of IFN-*γ* signaling, NK cells are able to kill MSCs, as an additional safety mechanism [85].

## 5. In Conclusion

Cell-based therapy employing MSCs has evolved as an interesting approach in the treatment of a wide range of autoimmune disorders as well as graft rejection, and a large number of clinical trials are currently ongoing. Although there are promising results the outcome has not fully met the expectations from preceding experiments in mouse models. This could be due to the differences observed between mechanisms of human and murine MSC immunoregulations. Furthermore, it is known that the immunomodulatory response changes depend on the given inflammatory environment. Increased knowledge about the complex crosstalk between MSCs and the immune system in humans could help to find clues about how to improve the therapeutic effect of MSCs. In the last years, cell priming has become an emerging research area in the field of MSCs immunomodulation. The encouraging results obtained aiming to enhance the immunoregulatory properties of MSCs render primed and genetically modified MSCs interesting alternatives worth being considered in future clinical trials. Additionally, analysis of data from clinical trials will be needed to optimize treatment dose, timing and frequency of administration. 

## Figures and Tables

**Table 1:** 

Pathology	Animal species	Target organ	Mechanism of action	WD	Ref.
Lupus erythematosus	Mouse	Bone marrow	Regeneration of hematopoietic niche	Sys	[22]
Rejection of HSC transplantation	Sheep	Hematopoietic compartment	Improve transplantation efficiency, increase hematopoiesis	Sys	[23]
Rejection of cutaneous grafts	Monkey	Skin	Inhibition of T cells	Sys	[24]
Ictus	Rat	Central nervous system	Secretion of neurotrophic factors	Sys	[25]
Acute renal failure	Rat	Kidneys	Secretion of trophic factors. Inhibition of proinflammatory cytokines	Sys	[26]
Diabetes	Mouse	Pancreas and renal glomeruli	Inhibition of macrophage infiltration	Sys	[27]
Diabetes	Mouse	Pancreas	Inhibition of *β* cell-specific T cells	Sys	[28]
Rejection of transplanted islets	Mouse	Kidney capsule	Inhibition of *β* cell-specific T cells	Loc	[29]
Rheumatoid arthritis	Mouse	Joints	Inhibition of secretion of proinflammatory cytokines and inhibition of T cells	Sys	[30]
Retinal degeneration	Rat	Eyes	Induction and secretion of trophic factors	Loc	[31]
Acute lung injury	Mouse	Lungs	Inhibition of secretion of proinflammatory cytokines	Sys	[32]
Acute lung injury	Mouse	Lungs	Inhibition of secretion of proinflammatory cytokines. Secretion of IL-10	Loc	[33]
Hepatic failure	Mouse	Liver	Inhibition of inflammatory infiltrate	CM	[34]
Experimental autoimmune encephalomyelitis	Rat	Central nervous system	Inhibition of myelin-specific T cells	Sys	[35]
Myocardial infarction	Rat	Heart	Secretion of trophic factor SFRP2	Loc	[36]
Ulcerative colitis	Mouse	Gut	Suppression of inflammatory infiltrates and cytokines. Increase of regulatory T cell activity	Sys	[37]
